# Segmental Arterial Mediolysis and Other Mimics of Medium Vessel Vasculitis: A Case and Review

**DOI:** 10.3390/jcm15103849

**Published:** 2026-05-16

**Authors:** Reena Yaman, Alejandro Arango Martinez, Carlos A. Padula, Andrew R. Lewis, Florentina Berianu, Benjamin Wang

**Affiliations:** 1 Division of Rheumatology, Mayo Clinic, Jacksonville, FL 32224, USA; 2 Department of Internal Medicine, Pontifical Bolivarian University, Medellín 050031, Colombia; 3 Department of Radiology, Mayo Clinic, Jacksonville, FL 32224, USA

**Keywords:** segmental arterial mediolysis, vasculopathy, vasculitis, diagnostic imaging

## Abstract

**Background:** Segmental arterial mediolysis (SAM) is a non-inflammatory vasculopathy that primarily affects the abdominal visceral arteries leading to hemorrhage, ischemia, or pseudoaneurysms. Its presentation can be mimicked by other vasculopathies including vasculitis involving the medium-sized blood vessels making it difficult to diagnose. **Case Presentation:** A 55-year-old woman presented with a two-hour history of sudden-onset, severe epigastric pain radiating to the chest. She was noted to be hypotensive with low hemoglobin 8.8 g/dL suspicious for a hemorrhagic cause. Her case was complicated by elevated international normalized ratio 3.7 in the setting of warfarin therapy for the mechanical mitral valve. The remainder of her complete blood count, complete metabolic panel, inflammatory markers, autoantibody serologies, and infectious testing were negative. Abdominal computed tomography angiogram revealed hemoperitoneum, bilateral renal infarctions, a large mesenteric hematoma, aneurysmal disease of the common hepatic and inferior mesenteric arteries, thrombosis and proximal dissection of the superior mesenteric artery, acute thrombosis of the left external iliac vein, and multiple sites of arterial extravasation from the pancreaticoduodenal artery and its branches. Mesenteric artery angiogram showed multivessel visceral artery aneurysms and stenoses characteristic of SAM for which she underwent transcatheter arterial embolization of the bleeding vascular bed. We provide a narrative literature review with a focus on common presentations and differentiating characteristics of vasculopathies that can involve medium-sized blood vessels. It is important to accurately diagnose SAM and its potential mimics as management strategies differ. **Conclusions:** SAM presents with medium vessel vasculopathy without vasculitis. Differentiation from mimics can be difficult but aided by familiarity of their characteristic findings and differentiating clinical characteristics.

## 1. Introduction

Segmental arterial mediolysis (SAM) was first described in 1976 by Slavin and González-Vitale [1,2]. It is an idiopathic non-inflammatory non-atherosclerotic vascular disease primarily affecting the abdominal visceral arteries [3]. Involvement of smaller vascular beds including the coronary, renal, iliac, pulmonary, and intracranial circulation occurs less frequently but has also been reported [3].

The epidemiology of SAM remains poorly defined, but its estimated incidence is approximately 1 in 100,000 individuals per year and has been reported to have a slight predominance in males [4]. Clinical manifestations vary depending on the affected vascular territory with common presentations including intra-abdominal hemorrhage, organ ischemia, or pseudoaneurysms [1,2]. Diagnosis is challenging as its clinical presentation, and angiographic findings can overlap with other vasculopathies such as fibromuscular dysplasia (FMD) or polyarteritis nodosa (PAN) [4]. Despite advancements in imaging techniques and therapeutic strategies, its pathophysiology remains incompletely understood, highlighting the need for further studies to improve comprehension and management. Given its unpredictable clinical course and the risk of severe complications, early recognition of SAM is crucial for optimizing treatment and improving patient outcomes. We present a case of SAM with accompanying narrative review to aid in highlighting the characteristics that help to differentiate it from other vasculopathies.

## 2. Case Presentation

A 55-year-old woman with a history of congenital atrioventricular canal defect, mechanical mitral valve replacement on chronic warfarin therapy, atrial flutter, and complete heart block with pacemaker in place presented to the emergency department with a two-hour history of sudden-onset severe epigastric pain radiating to the chest.

Upon arrival, she appeared diaphoretic and pale, with bradycardia and a persistently low mean arterial pressure of <50 mmHg. She denied symptoms suggestive of gastrointestinal bleeding, fever, chills, unintentional weight loss, rash, and symptoms of localized infection. Family history was noncontributory.

Initial laboratory tests were unremarkable except for moderate anemia, an international normalized ratio (INR) of 3.7, and elevated serum lactate. Coagulation studies were notable for prolonged prothrombin time (PT) and decreased protein C and protein S activity attributed to the patient’s known warfarin therapy. Laboratory studies are summarized in Table 1. Given the suspicion of hemorrhagic shock, aggressive resuscitation was initiated with intravenous fluids, massive transfusion protocol, and early vasopressor support.

Contrast-enhanced abdominal computed tomography (CT) revealed hemoperitoneum, bilateral renal infarctions, a large mesenteric hematoma, aneurysmal disease of the common hepatic and inferior mesenteric arteries, thrombosis and proximal dissection of the superior mesenteric artery, acute thrombosis of the left external iliac vein, and multiple sites of arterial extravasation from the pancreaticoduodenal artery and its branches. Variantyx connective tissue panel genetic testing returned negative.

**Figure 1:**
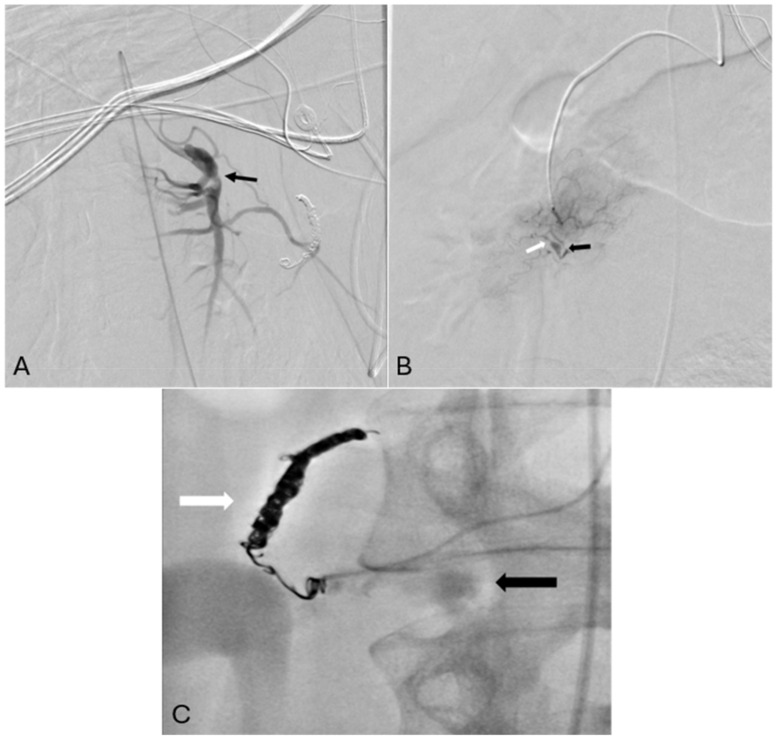
Angiography demonstrating findings consistent with segmental arterial mediolysis. (**A**) Superior mesenteric artery chronic dissection with aneurysmal degeneration (black arrow). (**B**) Superior pancreaticoduodenal artery multifocal stenoses (white arrow) and aneurysms (black arrow). (**C**) Active extravasation from the inferior pancreaticoduodenal duodenal artery (black arrow) with embolization coils in the superior pancreaticoduodenal artery (white arrow).

Given the hemodynamic instability and imaging findings, transcatheter arterial embolization of the bleeding vascular bed was performed, followed by continuous infusion of unfractionated heparin to replace warfarin. Angiography images are shown in Figure 1. Findings typical for SAM include multifocal dissections, aneurysms, and stenoses in the splanchnic and renal territories. In the absence of systemic symptoms, autoimmune disease markers, or alternative diagnoses in addition to characteristic imaging findings, a diagnosis of SAM was established.

**Figure 2:**
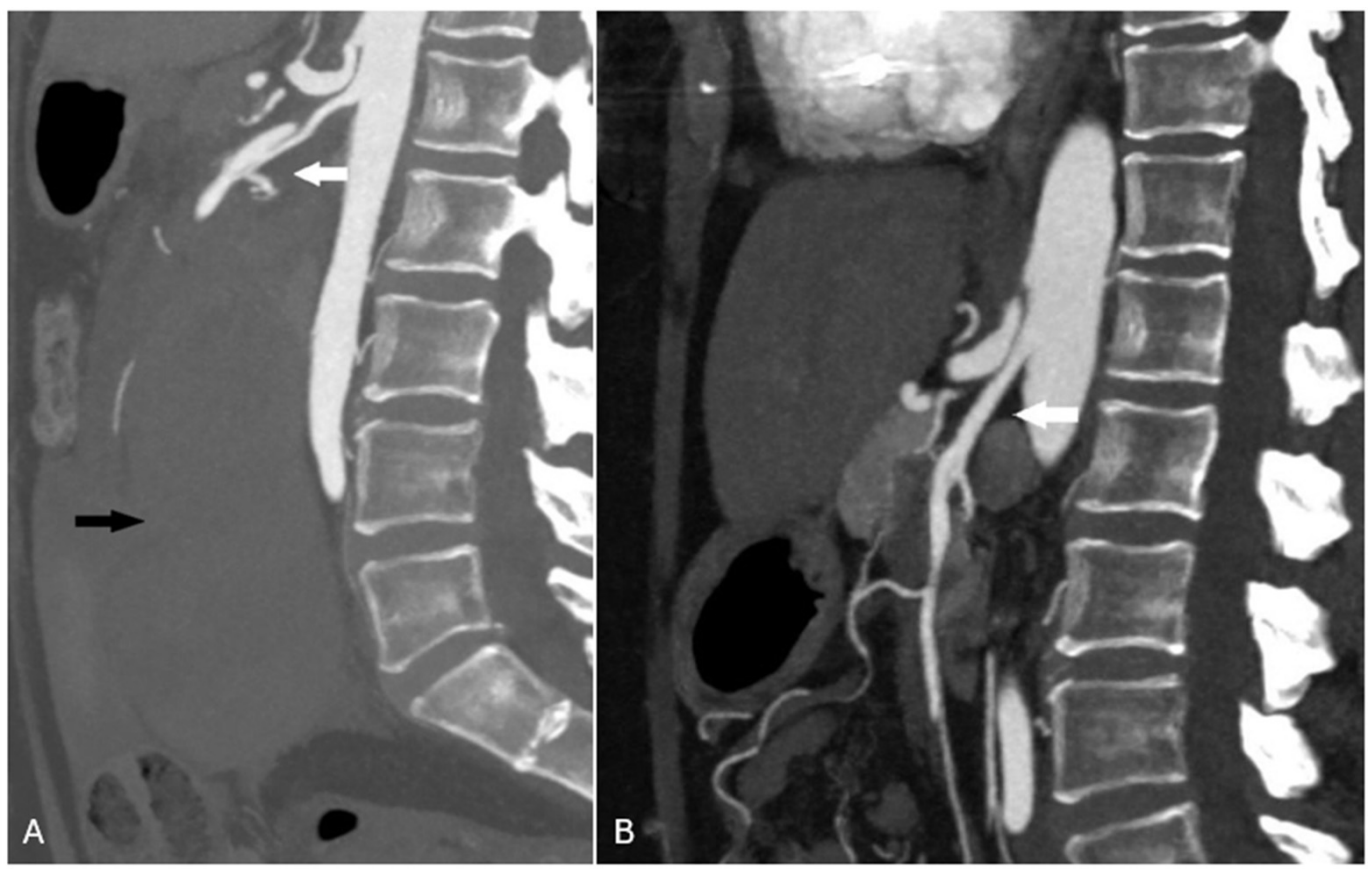
(**A**) Sagittal reformatted CT angiogram showing acute dissecting aneurysm of the proximal superior mesenteric artery (white arrow) and large 14 cm × 13 cm mesenteric hematoma with associated hemoperitoneum (black arrow). (**B**) 18-month follow-up saggital reformatted CT angiogram demonstrating healed superior mesenteric artery (white arrow).

Her disease was monitored using serial CT angiography with intravenous contrast. Findings at the 18-month follow-up demonstrated post-procedural changes given prior coil embolization with otherwise healed dissection of the superior mesenteric artery, healed kidney infarctions, and no residual identifiable SAM findings in the visceral arteries (Figure 2).

For transparency, accuracy, reduce risk of bias, and completeness in evidence-based case reports, the Supplementary File containing the CARE checklist is attached.

## 3. Discussion

Although the pathophysiological mechanisms of SAM remain incompletely elucidated, two key processes have been identified in vascular damage: an injury phase and a repair phase [5]. The injury phase is characterized by acute lysis of smooth muscle cells in the vascular wall leading to vacuole formation in the tunica media. This process predisposes vessels to transmural degeneration, weakening the vascular structure and increasing the risk of dissection, arterial tortuosity, intramural hematomas, and aneurysm formation [6]. The mediolytic phase is followed by the repair phase characterized by granulation tissue deposition. This process facilitates the extension of necrotic tissue resulting in vascular anatomical alterations including segmental stenosis or even complete arterial occlusion. In some cases, involvement extends to the internal elastic lamina leading to the formation of intimal plaques [6].

Slavin et al. initially proposed a pathophysiological mechanism mediated by vasospastic disease induced by circulating norepinephrine [2,7]. This hypothesis was supported by the description of SAM affecting the abdominal and coronary circulation in animal models following the administration of ractopamine (a β2-agonist). It has been postulated that ractopamine, similar to β2-agonists such as clenbuterol, may modulate the peripheral sympathetic nervous system, inducing norepinephrine release. This catecholamine, when released in cardiac sympathetic ganglia, activates β1-adrenergic receptors in cardiomyocytes, promoting myocardial apoptosis [2,7].

Additionally, norepinephrine synthesized in the varicosities of efferent nerves innervating the splanchnic arteries stimulates α1-receptors on the smooth muscle cell membrane. This activation induces intense and transient vasoconstriction followed by apoptosis or directly initiates apoptosis by disrupting the structural interface between the adventitia and the arterial media. This mechanism is believed to be responsible for the initial lesions in SAM, evidenced by fibrin deposits and hemorrhages at the adventitia-media junction [2,7].

Beyond arterial involvement, venous damage in SAM has also been described—referred to as venous angiopathy. This typically manifests in the abdominal circulation, affecting territories adjacent to large-caliber arteries with findings consistent with SAM [5]. These lesions are often segmental and may partially or completely involve the vessel circumference [5]. Mediolysis in this context results from cellular membrane dissolution, followed by an inflammatory process causing venous edema, leading to the separation of the muscular layer, disrupting its contact with the intima, and contributing to luminal narrowing [8]. Medial muscle loss results in irregular contours of the external medial wall (“moth-eaten” appearance), or in cases of transmedial involvement with intimal loss, defects may lead to perivenous hemorrhages and/or luminal thrombosis formation [9]. Once the medial loss areas undergo fibrotic repair, the resulting venous lesions closely resemble fibromuscular dysplasia [9].

The clinical manifestations of SAM vary according to the affected vascular beds. In abdominal involvement, symptoms often include pain, distension, a rapid decline in hematocrit, and hypovolemic shock secondary to peritoneal or retroperitoneal hemorrhage [6]. Bleeding usually originates from a single artery but can involve multiple major abdominal vessels, most commonly affecting the splenic, hepatic, and distal renal arteries [10]. In some cases, smaller branches, such as pancreatic, intrahepatic, and submucosal intestinal arteries, may be involved [10]. Abdominal SAM may also present without massive hemorrhage, instead manifesting as organ damage secondary to arterial thrombosis. Hematuria due to renal cortical infarctions can be an initial finding, along with intestinal ischemic lesions caused by progressive arterial narrowing [10]. Concurrent thrombosis and bleeding is thus characteristic of SAM given the endothelial abnormalities and arterial wall damage, respectively. Acquired coaguopathy, as seen in this case, could theoretically amplify the hemorrhagic complications of the disease. Interestingly, anticoagulation has been reported as a potential treatment for SAM demonstrating the interesting yet poorly elucidated paradox of this disease [11].

The management of SAM depends on disease severity. In cases of hemorrhagic shock, hemodynamic stabilization is essential in addition to avoidance of α1-adrenergic agonists, which can exacerbate the disease and induce new lesions. Emergency surgery may be required for resection, ligation, or bypass of bleeding aneurysms. As a less invasive alternative, transcatheter embolization provides immediate hemorrhage control, utilizing N-butyl cyanoacrylate in cases where arterial tortuosity precludes microcatheter use. Additionally, the therapeutic potential of endothelin-1 (ET-1) antagonists is under investigation [12]. Although various interventional procedures can be necessary for the management of vasculopathies, the main differentiator for the management of vasculitis compared to non-vasculitis mimics is the need for immunosuppression [11,13]. The entire scope of treatment of SAM and its mimics is beyond the scope of the current review.

Diagnosing SAM is extremely challenging and the gold standard for diagnosis remains histopathological analysis [14]. However, obtaining tissue samples is often unfeasible as vessels are fragile during the active phase of the disease, posing a high risk of bleeding, or are located deep within the abdominal cavity [15]. This case demonstrates this limitation as diagnosis was dependent on radiologic findings given the absence of available histologic specimens. In the late, stable phase, pathology studies only reveal residual fibrosis without specific findings to confirm the diagnosis. For this reason, SAM diagnosis is primarily based on clinical presentation and radiologic findings. Nevertheless, a wide range of conditions can mimic this entity, making differential diagnosis and exclusion of potential mimics critically important [16]. As such, proposed diagnostic criteria include angiographic findings consistent with SAM in addition to “negative criteria” focused on excluding mimics [17].

The first step in evaluating SAM is ruling out vasculitis by identifying underlying causes such as autoimmune diseases, infections, or drug exposures [16]. Among the vasculitides affecting the medium-sized vessels, polyarteritis nodosa (PAN) is the primary condition to consider due to its necrotizing and inflammatory nature, affecting the tunica media. PAN can mimic SAM, particularly in its early phase, as it involves visceral vasculature and shares radiographic findings such as aneurysm formation [18]. Other vasculitides, such as giant cell arteritis and Behçet’s disease, although less common in visceral arteries, can also affect medium-sized vessels and present with thrombosis or aneurysms [19]. Systemic vasculitis is often accompanied by nonspecific symptoms such as fatigue, fever, unintentional weight loss, or cutaneous manifestations. Another distinguishing feature is elevated inflammatory markers, typically present in systemic vasculitis [20]. Large vessel vasculitis affecting the medium vessels can be differentiated similarly and has more diffuse imaging findings affecting the characteristic large vessels [4,16].

Multifocal fibromuscular dysplasia (FMD) and SAM may share overlapping angiographic and clinical features, complicating differentiation [21]. Multifocal FMD is characterized by the classic “string-of-beads” pattern, with alternating stenoses and dilations, predominantly affecting the renal, iliac, and cervical arteries. Its course is chronic, with symptoms related to stenosis, such as renovascular hypertension or pulsatile tinnitus [22]. In contrast, SAM is an acute process primarily affecting celiac and mesenteric arteries, with rapid progression to aneurysms and a high risk of intra-abdominal bleeding. Visceral involvement in FMD is rare and usually asymptomatic [23].

Genetic non-inflammatory connective tissue disorders such as Marfan Syndrome tend to have other characteristic clinical findings and potential for genetic testing to aid with diagnosis [16]. Differentiating SAM from atherosclerotic aneurysms is complex, as many patients with SAM have preexisting atherosclerosis. However, atherosclerosis generally progresses slowly. In imaging studies, the presence of calcifications and plaques suggests atherosclerosis, whereas SAM exhibits a segmental pattern without calcifications [24].

Given the lack of available laboratory biomarkers, and frequent absence of biopsy specimens, diagnosis of these entities is largely based on clinical presentation and radiologic findings. Review of abdominal CT and MRI imaging with concern for presence of medium-vessel vasculitis, showed sensitivity 67% and specificity of 92% when compared to final clinical diagnosis. False positive results included SAM and FMD as PAN mimics [25]. Table 2 summarizes the characteristic clinical, radiologic, and laboratory findings seen in SAM and its common mimics PAN, FMD, genetic connective tissue disorders, and large vessel vasculitis.

## 4. Conclusions

We present a case of SAM presenting with hemoperitoneum complicated by comorbid coagulopathy in the setting of chronic warfarin therapy. Diagnosis of SAM can be challenging and differentiation from entities that can cause medium vessel vasculopathy, including vasculitis, can be aided by familiarity with characteristic clinical and radiologic findings of common mimics.

## Figures and Tables

**Table 1:** Laboratory results including blood counts on presentation and subsequent immunologic and infectious testing.

Parameter	Patient Value	Reference Range
Hemoglobin	8.8 g/dL	11.5–15.0 g/dL
Hematocrit	26.9%	35.5–44.9%
MCV	90.6 fL	78.2–97.9 fL
MCH	29.6 pg	25.4–32.7 pg
Platelet count	242 × 10^9^/L	157–371 × 10^9^/L
Leukocyte count	9.3 × 10^9^/L	3.4–9.6 × 10^9^/L
Neutrophil count	7.16 × 10^9^/L	1.56–6.45 × 10^9^/L
Lymphocyte count	1.56 × 10^9^/L	0.95–3.07 × 10^9^/L
Monocyte count	0.41 × 10^9^/L	0.26–0.81 × 10^9^/L
Eosinophil count	0.05 × 10^9^/L	0.03–0.48 × 10^9^/L
Basophil count	0.02 × 10^9^/L	0.01–0.08 × 10^9^/L
PT	42 s	9.4–12.5 s
INR	3.7	0.9–1.1
aPTT	33 s	25–37 s
Fibrinogen	186 mg/dL	200–393 mg/dL
D-dimer	21,251 ng/mL	≤500 ng/mL
Antithrombin activity	95%	80–130%
Protein C activity	47%	70–150%
Free protein S antigen	43%	65–160%
Total protein S antigen	82%	80–160%
PT correction with 1:1 mixing study	12.6 s	9.4–12.5 s
ESR	<1 mm/1 h	0–29 mm/1 h
CRP	4 mg/L	<5 mg/L
ANA	Negative	Negative
SS-A/Ro	<0.2 U/mL	<25 U/mL
SS-B/La	<0.2 U/mL	<25 U/mL
Sm	<0.2 U/mL	<25 U/mL
RNP	<0.2 U/mL	<25 U/mL
Scl-70	<0.2 U/mL	<25 U/mL
Jo-1	Negative	Negative
C3	110 mg/dL	88–201 mg/dL
C4	25 mg/dL	15–45 mg/dL
Rheumatoid factor	<15 UI/mL	0–30 IU/mL
Myeloperoxidase	<0.2 UI/mL	<20 U/mL
Proteinase 3	<0.2 UI/mL	<20 U/mL
Hepatitis B Surface Antigen	Negative	Negative
Hepatitis B Core Antibody	Negative	Negative
Hepatitis C Virus Antibody	Negative	Negative
HIV	Non-reactive	Non-reactive

Abbreviations: aPTT, activated partial thormboplastin time; ANA, antinuclear antibody; CRP, C-reactive protein; ESR, erythrocyte sedimentation rate; HIV, human immunodeficiency virus; INR, international normalized ratio; MCH, mean corpuscular hemoglobin; MCV, mean corpuscular volume; PT, prothrombin time; Ro/SS-A, Sjögren’s syndrome-related antibody A; La/SS-B, Sjögren’s syndrome-related antibody B; RNP, ribonucleoprotein antibody; Sm, Smith antibody; Scl-70, topoisomerase I antibody; Jo-1, histidyl-tRNA synthetase antibody; C3, complement component 3; C4, complement component 4.

**Table 2:** Most common and/or characteristic clinical, radiologic, and laboratory findings of SAM and its potential mimics. Adapted from references [4,16,25].

	Segmental Arterial Mediolysis	Polyarteritis Nodosa	Fibromuscular Dysplasia	Genetic Connective Tissue Disorder	Large Vessel Vasculitis
**Gender predominance**	None	Male	Female	Equal	Female
**Radiologic findings**	Dissection and aneurysms without significant wall thickening, high rates of rupture	Wall thickening and stenosis with characteristic small aneurysms at arterial branchpoints	Circumferential stenosis, characteristic “string of beads” appearance	Dissection and aneurysms	Narrowing or occlusion
**Affected arteries**	Mesenteric (commonly SMA, hepatic, celiac)	Mesenteric	Renal and cerebrovascular	Multisystem	Aorta and its branches
**Laboratory findings**	None	Elevated inflammatory markers	None	Characteristic genetic mutations	Elevated inflammatory markers
**Treatment**	Medical optimization, surveillance, and intervention as indicated	Immunosuppressive therapy and intervention as indicated	Medical optimization, surveillance, and intervention as indicated	Medical optimization, surveillance, and intervention as indicated	Immunosuppressive therapy and intervention as indicated

## Data Availability

No new data were created.

## Supplementary Materials

The following supporting information can be downloaded at: https://www.mdpi.com/article/10.3390/jcm15103849/s1, Table S1: CARE Checklist of information to include when writing a case report.
